# First Reported Case of Methicillin-Resistant *Staphylococcus aureus* Vertebral Osteomyelitis with Multiple Spinal and Paraspinal Abscesses Associated with Acupuncture

**DOI:** 10.1155/2015/524241

**Published:** 2015-07-15

**Authors:** Sandeep Singh Lubana, Mostafa Alfishawy, Navdeep Singh, Debra J. Brennessel

**Affiliations:** ^1^Icahn School of Medicine at Mount Sinai, Queens Hospital Center, 82-68 164th Street, Queens, NY 11432, USA; ^2^Department of Ambulatory Care Program, Internal Medicine Residency Program, Icahn School of Medicine at Mount Sinai, Queens Hospital Center, 82-68 164th Street, Queens, NY 11432, USA

## Abstract

Acupuncture is one of the oldest medical procedures in the world and originated in China about 2,000 years ago. Acupuncture is a form of complementary medicine and has gained popularity worldwide in the last few decades. It is mainly used for the treatment of chronic pain. Acupuncture is usually considered a safe procedure but has been reported to cause serious complications including death. It has been associated with transmission of many viruses and bacteria. Two cases of Methicillin-Resistant *Staphylococcus aureus* have been reported recently following acupuncture therapy. We are reporting a case of a 57-year-old Korean female who developed vertebral osteomyelitis and intraspinal and paraspinal abscesses as a complication of acupuncture. Blood cultures, skin lesion culture, and body fluid culture yielded Methicillin-Resistant *Staphylococcus aureus* (MRSA). Good anatomical and medical knowledge, good hygiene standards, and proper acupuncture techniques should be followed to prevent the complications. Acupuncturists should consistently review the infection control guidelines to acupuncture. This case should raise awareness of such condition and hazards of presumably benign procedures such as acupuncture.

## 1. Introduction

Acupuncture in Latin words means “acus” (needle) and “puncture” (penetration). Acupuncture is one of the oldest medical procedures in the world and originated in China about 2,000 years ago. In United States it appeared in the early 18th century but gained popularity after 1971 when New York Times journalist, James Reston, reported his experience with acupuncture. He was visiting China for President Nixon's visit. He had an emergency appendectomy in China and was given acupuncture for postoperative analgesia [1]. Acupuncture is mainly used for the treatment of chronic pain such as back pain, neck pain, and joint pain and for conditions like fatigue, insomnia, and depression. However, acupuncture, presumably a benign procedure, is related to the transmission of infectious agents and can cause devastating complications. We are reporting a first case of community acquired Methicillin-Resistant* Staphylococcus aureus* (MRSA) acute osteomyelitis with intraspinal and multiple paraspinal abscesses related to acupuncture.

## 2. Case Report

A 57-year-old Korean female presented with complaints of two-day history of nausea, vomiting, and generalized weakness along with subjective fever and night sweats. The patient also complained of intermittent chronic back pain for years that worsened over last week and made her go to have acupuncture therapy for the first time two days priorly for her chronic back pain but unfortunately pain got worse. Her physical examination revealed tenderness in the lumbosacral region without any redness or erythema. Laboratory tests showed normal white count with bands of 34% (normal range: 0–5%). Chest X-ray showed patchy opacities in the bilateral lower lung fields. Blood cultures were drawn and the patient was started on broad-spectrum antibiotics.

The next day after admission the patient developed urinary retention. Computerized Tomography (CT) scan of the lumbar spine was done to rule out disc herniation, which instead showed enlargement of the right psoas and quadratus lumborum muscles with induration of paraspinal soft tissue. Magnetic Resonance Imaging (MRI) of the lumbar spine showed fluid in the soft tissues posteriorly, which was reported as nonspecific but could be related to acupuncture.

**Figure 1:**
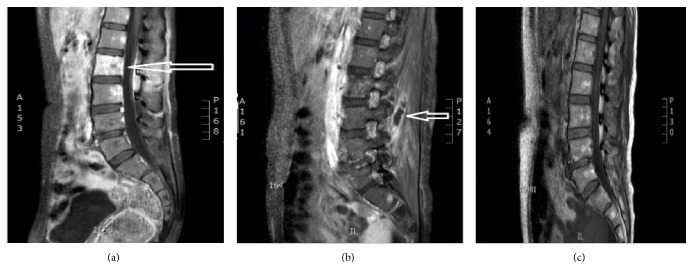
MRI lumbar spine. (a) MRI of the lumbar spine with contrast showing L2 vertebral osteomyelitis. (b) MRI of the lumbar spine showing paraspinal abscess. (c) Follow-up MRI of the lumbar spine showing resolution of the osteomyelitis and the paraspinal abscesses.

Blood cultures came back positive for MRSA and only Vancomycin was continued. The patient developed multiple 2 mm new skin pustules over her legs and cultures from the skin lesions were also positive for MRSA. Transthoracic and transesophageal echocardiogram failed to show any vegetation. A chest CT and abdominal MRI were done to discover the occult source of infection. The chest CT showed multiple necrotizing nodules suggestive of septic emboli. The abdomen MRI showed fluid collection posterolateral to the right psoas muscle, which was likely due to abscess formation. Multiple new small microabscesses in the paraspinal muscles and early osteomyelitis of the L2 vertebral body were also reported (Figures 1(a) and 1(b)). A new small (0.6 × 0.5 cm) intraspinal fluid collection was also seen at the L2 vertebral body. CT-guided aspiration of the right paraspinal abscess was done and cultures were sent, which came back positive for MRSA as well. The patient was continued on Vancomycin.

MRI and CT of the lumbar spine after four weeks of antibiotic therapy again demonstrated osteomyelitis of the L2 vertebra. The paraspinal fluid collections demonstrated interval decrease in size and resolution of the intraspinal fluid collection. The patient reported improvement of back pain and was discharged on a 12-week course of intravenous Vancomycin. An MRI of the lumbar spine (Figure 1(c)) eight weeks following antibiotic therapy showed significant improvement of osteomyelitis and almost complete resolution of abscesses. The delayed CT scan showed complete resolution of abscesses. During a follow-up visit the patient stated that her back pain had resolved.

## 3. Discussion

Acupuncture is gaining popularity as a common therapeutic procedure for chronic pain control in the United States. However, due to failure to follow infection control guidelines for acupuncture therapy (Australian Acupuncture Association Limited, 1997) [10], there has been transmission of various infectious agents (Table 1). A review of the literature revealed that infectious agents such as hepatitis B and hepatitis C virus in 1988 and 1993 [11, 12], Human Immunodeficiency Virus in 1989 [13], Mycobacteria in 2001 and 2002 [14, 15], Methicillin Sensitive* Staphylococcus aureus* (MSSA) in 1997, 2002, 2003, 2004, and 2006 [2–7], and Methicillin-Resistant* Staphylococcus aureus* (MRSA) in 2008 [8, 9] were reported to have been transmitted by acupuncture. Now in 2015 we are reporting a first case of acupuncture associated MRSA acute vertebral osteomyelitis with intraspinal and multiple paraspinal abscesses.

Along with infectious complications, acupuncture is also known to be associated with mechanical organ injuries. Three deaths have been reported in which acupuncture was claimed as the cause, with one patient who died from endocarditis, another from bilateral pneumothorax, and a third due to severe asthma during acupuncture therapy. The adverse effects of acupuncture can be prevented with sound anatomical and medical knowledge, proper hygiene practices, and adequate acupuncture education [16].

Vertebral osteomyelitis is classified as being acute, subacute, or chronic. Acute osteomyelitis develops in a few days or weeks while subacute or chronic occurs over weeks to months before treatment is initiated. Vertebral osteomyelitis occurs via hematogenous, direct inoculation, or contagious transmission. The lumbar spine is most commonly involved (58%), followed by the thoracic spine (30%) and the cervical spine (11%) [17].

Patients with vertebral osteomyelitis usually present with back pain and fever and may have some form of neurologic deficit. MRI has a high sensitivity (96%) and specificity (92%) for diagnosing vertebral osteomyelitis [18]. However, response to treatment is determined by clinical assessment and inflammatory markers since radiologic improvement by MRI is delayed and always follows clinical improvement [19]. Follow-up MRI is only indicated when there are no signs of clinical improvement after four weeks of therapy or an abscess is suspected [20].

A high index of clinical suspicion is required for vertebral osteomyelitis since the presentation can be very vague. Early imaging studies and empiric antibiotic therapy covering MRSA should be initiated to prevent serious complications, since* Staphylococcus aureus* is the most common organism causing vertebral osteomyelitis [21]. Treatment duration for osteomyelitis is generally recommended for six weeks but a longer course should be considered in complicated infections [22]. In the present case, the patient received parenteral Vancomycin for three months and CT-guided aspiration of paraspinal abscess which resulted in a favorable outcome.

In the present case, back pain worsened over weeks which is the natural course of mechanical low back pain but having acute worsening after acupuncture directly suggests that bacterial inoculation at the time of procedure which was further complicated by bacteremia although there was no local skin abscess at the site of acupuncture temporal correlation is evident. The patient had been in the United States for years and this was her first hospitalization and she did not have any other medical problem suggesting that she could be carrier for MRSA so it was assumed that this infection was acquired from acupuncture as reported from previous literature.

To best of our knowledge, only two cases of MRSA associated with acupuncture have been reported in the literature to date. Lee et al. [8] reported acupuncture associated MRSA necrotizing aortitis in April 2008 and Woo et al. [9] reported MRSA causing septic arthritis following acupuncture in August 2008. We are reporting a third case of MRSA associated with acupuncture. However, this is the first case of MRSA associated vertebral osteomyelitis with spinal and paraspinal abscesses.

## 4. Conclusion

Vertebral osteomyelitis and/or abscess should be considered in the differential diagnosis in a patient presenting with worsening back pain following acupuncture therapy. Diagnoses of vertebral osteomyelitis can easily be missed or delayed due to its vague presentation. Careful history taking and early initiation of therapy is of paramount importance for a favorable outcome. Acupuncturists should possess sound anatomical and medical knowledge and adhere to proper hygiene standards and techniques with consistent review of the infection control guidelines pertaining to acupuncture to prevent the devastating complications and significant financial expenditure due to prolonged hospitalization. This case raises awareness of the possible hazards of presumably benign procedures such as acupuncture.

## Figures and Tables

**Table 1:** Infectious complications related to acupuncture (MSSA and MRSA).

	References	Gender/age	Reason for acupuncture	Joint(s)/area(s) involved	Causative organism	Site(s) of positive culture	Treatment/duration of the therapy	Outcome
1	Kirschenbaum and Rizzo (1997) [2]	M/76	Intermittent left sided shoulder pain-several months duration	Left shoulder joint with diffuse swelling & tenderness	MSSA	Thick purulent synovial fluid	Arthrotomy, surgical debridement, partial synovectomy, and irrigation and oxacillin for 6 weeks	Remission

2	Laing et al. (2002) [3]	F/45	For faster recovery from (Schatzker type II) tibial plateau fracture	Left knee	MSSA	Skin swabs and joint fluid aspirate	Arthroscopic washout and 7-week antibiotic therapy	Decreased range of knee motion (0–120 degrees)

3	Woo et al. (2003) [4]	—	Low back pain	Back midline	MSSA	Subcutaneous abscess	Surgical debridement and drainage and 5-week cloxacillin therapy	Remission

4	Daivajna et al. (2004) [5]	M/48	Chronic low back pain	L5/S1 facet joint and lower paraspinal region	MSSA	CT-guided biopsy and joint aspirate	Surgical debridement and 6-week antibiotic therapy	Full range of movement of lumbar spine

5	Chen et al. (2004) [6]	M/44	Chronic nuchal and subscapular pain	Mass lesion involving C6–T1 spine causing cord compression	MSSA	Pus obtained at surgery	Laminectomy from C6 through T1, drainage of multiple subdural abscess, and copious irrigation and 6-week course of oxacillin and rifampin	Mild residual left hand paresis

6	Seeley and Chambers (2006) [7]	M/31	Persistent right hip pain	Right obturator externus and adductor muscles	MSSA	Blood culture, thigh abscess aspirate	CT-guided abscess drainage and 5-week antibiotic therapy	Remission

7	Lee et al. (2008) [8]	M/79	Unknown	Abdominal aorta	MRSA	Aortic wall and atheroma	Emergent exploratory laparotomy, resection of the infected aorta, debridement, and axillary to bifemoral bypass and prolonged antibiotic therapy	Remission

8	Woo et al. (2009) [9]	F/43	Knee pain	Left knee	MRSA	Synovial fluid/tissue	Arthrotomy, synovectomy, and 6-month antibiotic therapy	Remission

9	Present case	F/57	Chronic back pain	Lumbar spine, spinal and paraspinal muscles	MRSA	Blood culture, skin lesion culture, paraspinal abscess aspirate culture	CT-guided abscess drainage and 12-week intravenous Vancomycin therapy	Remission

M: male, F: female.
